# Air displacement plethysmography (pea pod) in full-term and pre-term infants: a comprehensive review of accuracy, reproducibility, and practical challenges

**DOI:** 10.1186/s40748-018-0079-z

**Published:** 2018-06-20

**Authors:** Hajar Mazahery, Pamela R. von Hurst, Christopher J. D. McKinlay, Barbara E. Cormack, Cathryn A. Conlon

**Affiliations:** 1 College of Health, Massey University, Auckland, 0745 New Zealand; 2 Kidz First Neonatal Care, Counties Manukau Health, Auckland, New Zealand; 3 Liggins Institute and Department of Paediatrics: Child and Youth Health, University of Auckland, Auckland, New Zealand; 4 Starship Children’s Hospital, Auckland District Health Board, Auckland, New Zealand

**Keywords:** Air displacement plethysmography, ADP, Pea pod, Infant, Pre-term, Full-term, Body composition

## Abstract

Air displacement plethysmography (ADP) has been widely utilised to track body composition because it is considered to be practical, reliable, and valid. Pea Pod is the infant version of ADP that accommodates infants up to the age of 6 months and has been widely utilised to assess the body composition of full-term infants, and more recently pre-term infants. The primary goal of this comprehensive review is to 1) discuss the accuracy/reproducibility of Pea Pod in both full- and pre-term infants, 2) highlight and discuss practical challenges and potential sources of measurement errors in relation to Pea Pod operating principles, and 3) make suggestions for future research direction to overcome the identified limitations.

## Background

Early postnatal growth is a major determinant of short- and long-term health outcomes, for both full-term and pre-term infants. The significance of neonatal nutritional management and postnatal growth lies in their effect on infant survival, neurodevelopment and later metabolic function. Postnatal faltering growth is associated with neurodevelopment impairment, especially in preterm infants, and increased risk of cardiometabolic disease, particularly after fetal growth restriction [1, 2]. Rapid postnatal growth is also associated with increased risk of metabolic syndrome or its determinants [3–7]. These complex effects are influenced by differential growth in soft tissue compartments, namely, fat mass (FM) and lean mass (LM). Growth that involves relatively greater gains in LM appears to be protective for brain development [8, 9] and metabolic function [9], whereas accelerated gain in FM, especially centrally and in the first 6 months, is associated with childhood obesity [10]. Preterm infants appear to be particularly susceptible to both postnatal growth failure and central fat deposition [5]. These observations highlight the importance of assessing body composition and not just total mass when monitoring growth in infants.

There are a number of techniques available to measure body composition in infants, including dual energy X-ray absorptiometry (DXA), bioelectrical impedance analysis (BIA), isotope dilution, magnetic resonance imaging (MRI), and air displacement plethysmography (ADP) [11]. Local FM deposits may also be quantified by ultrasound [12]. ADP has gained popularity due to availability, feasibility and acceptability to parents [13]. ADP utilizes the inverse relationship between pressure and volume in two enclosed chambers – employing the gas law of Boyle and Poisson – that allows for the calculation of body density [14]. A two-compartment model is derived assuming a fixed density of fat (0.9007 g/ml) [15] and from age- and sex-specific estimates of fat free mass (FFM) density.

There are two commercially available ADP body composition systems; one for adults (Bod Pod) and one for infants (Pea Pod) (Cosmed, Rome, Italy). While Bod Pod houses both children aged > 2 years old and adults, Pea Pod accommodates infants from birth until approximately 6 months of age (body weight ≤ 10 kg). Recently, Pea Pod has also been employed to assess the body composition of pre-term infants from 30 weeks’ gestation, and may be a viable method for close monitoring of growth in these infants.

Since the introduction of Pea Pod in 2003 [14, 16, 17] and its validation in 2004 [18], a large number of technical and clinical research papers utilising Pea Pod have been published. Pea Pod has also been included in several recent reviews on body composition, including the utility of ADP technique from infancy to adulthood [19], changes in body composition over the first six months after birth utilising different assessment methods [4, 20], and a comparison of different body composition assessment tools in infancy [11]. However, to our knowledge, no comprehensive review of the use of Pea Pod in both full- and pre-term infants is available.

This review will focus exclusively on studies that have assessed body composition of infants using Pea Pod, including both full- and pre-term infants. We will summarise the published papers, with sections devoted to its accuracy relative to criterion methods, reproducibility, and to practical challenges associated with the use of Pea Pod in clinical settings and studies. From there, we will make suggestions for future research directions to address any identified limitations. At the outset, we present an overview of body composition measures, including assessment of accuracy and reproducibility, following which we describe the results of the review.

### Body composition measures

Body composition may be described using two-, three- and four-compartment models. The two-compartment model [21] divides body weight into FM and FFM (with assumptions made regarding the density of FM and FFM) and ignores the interindividual variation in FFM composition. The three-compartment model [22] is based on measurements of body density and total body water, and assumes a constant mineral to protein ratio in the dry FFM and makes no assumptions about the hydration of FFM. The four-compartment model [23] divides the body weight into fat, water, mineral, and protein, and is the most robust and sensitive to interindividual variability in the composition of FFM. Accordingly, the four-compartment model has less error than other models.

Obesity is defined as an excess of body fat that adversely affects health [20]. Body mass index (BMI, kg/m^2^) has long been used in clinical practice and epidemiological studies as an indicator of adiposity. However, BMI has been shown to have inconsistent relationship with disease risk and mortality. For example, Nagayama et al. [24] reported an inverse relationship between BMI and cardiometabolic fitness in healthy Japanese adults, whereas Flegal et al. [25] showed that all-cause mortality rate was lower in overweight than normal weight individuals.

This inconsistency may reflect the variable relationship between BMI and adiposity, with the latter being more closely related to disease risk [26]. For example, in a study of 709 adults aged 20–94 years (of both sexes and different ethnicities), Gallagher et al. [26] showed that BMI alone explained only 25% of the between-individual variability in adiposity. Further, Zeng et al. [27] showed that adiposity, but not BMI, was independently associated with cardiovascular risk factors. Even in normal weight individuals, higher adiposity has been shown to be associated with adverse cardiometabolic health outcomes, such as metabolic syndrome, dyslipidemia, hypertension, and cardiovascular disease [28].

In children, increases in BMI can reflect increases in FFM rather than increases in FM due to the rapid growth of musculoskeletal tissues [29]. A recent systematic review and meta-analysis found that BMI had high specificity but low sensitivity for detection of excess adiposity in children, and thus BMI failed to identify > 25% of children with excess FM [30]. Hetherington-Rauth et al. [31] compared indirect measures of adiposity (including BMI) with direct measures (DXA FM% and Fat Mass Index, FMI) in relation to cardiometabolic risk factors in children. Although the authors reported that anthropometric measures performed as well as DXA measures did, addition of direct measures of adiposity to indirect measures may be advantageous for predicting cardiometabolic risk factors such as insulin resistance [31], a finding reported by others [29, 32]. Similarly, in both full-term and pre-term infants, BMI was shown to have a weak association with FM% (defined as FM/weight*100) [33, 34], though the relationships were slightly stronger in full-term infants [35]. Thus, in infants and children, BMI appears to be a poor measure of adiposity, and “normal” BMI may still be associated with excess fat mass for size, particularly if LM is low [36].

Adiposity is often referenced to total mass (e.g, whole-body FM%) but this is statistically problematic given that fat mass is included in both numerator and denominator [37]. As body fat mass increases, FM% does not increase proportionately (depending on relative growth in FFM), resulting in underestimation of adiposity. The growth velocity of the different soft tissue compartments may be variably affected by nutritional interventions and factors such as age, sex, pubertal status, and ethnicity [38]. To overcome these problems, Van-Itallile [39] advocated referencing of FM and FFM to height squared, giving a FM index (FMI) and FFM index (FFMI), which in adults effectively normalises soft tissue mass for height and allows for comparison between individuals of different size.

A similar approach has also been recommended in children [40–42], although second order indices may not render soft tissue mass independent of size [40]. This is particularly important when comparing body composition between infants of differing length, especially in pre-term infants whose lower absolute FFM is partly due to their shorter body length. Two studies have reported centiles for FMI and FFMI in the first 6 months after birth, one using DXA in term and preterm infants [35] and the other using Pea Pod in term infants [43]. However, neither study reported if these indices remained correlated with length, so it is unclear if the measures used adequately accounted for differences in body size. This problem can be overcome with the use of sex-specific FM and FFM z-scores for length [40].

### Accuracy and reproducibility

Evaluation of any quantitative assessment technique, including body composition systems, comprises of an examination of its accuracy and reproducibility. Accuracy should be determined against an established criterion method that measures the same physical properties. Hydrostatic weighing is commonly used to validate ADP techniques, but this is not suitable for infants because it requires submerging them in water. Thus, for Pea Pod alternative techniques have been used, including tissue phantoms [17], deuterium dilution [18], DXA [44] and combined techniques with multi-compartment models [45].

Tissue phantoms are constructed from animal tissue using fat and muscle compartments, and the composition is usually determined from chemical analysis. DXA is a non-invasive technique that provides whole-body and regional estimates of three main components: bone mineral, bone-free FFM and FM [46]. DXA relies on the differential absorption of x-rays of two different energies and bone edge detection algorithms to calculate bone area and mineral content, and FM and FFM [46]. Isotope dilution, a standard technique for measuring total body water, involves administration of a known dose of isotope to a subject, allowing the isotope to equilibrate and enrich within the body water compartments (including saliva, urine or blood), and then measuring the isotope amount [47]. Isotope serves as a marker for total body water, from which FFM (using a specific hydration factor) and FM can be calculated [46].

Statistically, accuracy is commonly assessed by Bland-Altman analysis, [48] which relates the differences between two techniques to the mean value of the measures. Bias is the mean difference between the techniques and 95% of limits of agreement indicate the possible range of differences between the techniques. Reproducibility of paired measures can also be assessed by Bland-Altman analysis or for multiple measures of the same subject or phantom, by the coefficient of variation, which is the standard deviation as a percentage of the subject or phantom mean value.

## Method

We performed a literature search covering studies published in PubMed from 2003 up to September 2017. Because the first evaluation study of Pea Pod was published in 2003 we limited our search strategy to include papers published after 2003 to September 2017. Key search terms included “air displacement plethysmography” OR “Pea Pod” AND (infant OR infancy). Reviews and non-English papers were excluded. We also excluded papers that included non-human species. However, those studies that used animal species to investigate the accuracy/reproducibility of Pea Pod were included.

## Results

**Fig. 1:**
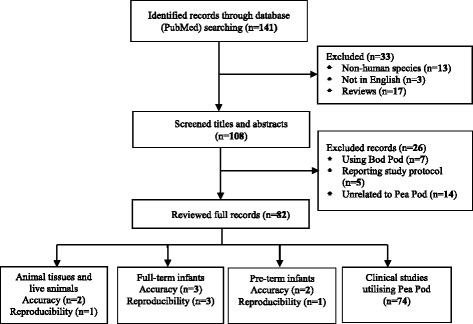
Flow diagram of study selection process

Our literature search identified 141 papers, including 13 non-human studies, three non-English papers, and 17 reviews, leaving 108 papers for title and abstract checking. Of these, 26 papers were excluded because they referred to Bod Pod (*n* = 7), reported a study protocol (*n* = 5), or were unrelated to Pea Pod (*n* = 14). Thus 82 papers were included in the final review, two of which were accuracy studies using animal tissues and live animals, four were accuracy/reproducibility studies in full-term infants, two were accuracy/reproducibility studies in pre-term infants, and 74 were clinical studies using Pea Pod to assess the body composition of either full-term infants, pre-term infants or both (Fig. 1). Characteristics of included validation studies are presented in Table 1.

**Table 1:** Accuracy and reproducibility studies of Pea Pod in full-term and preterm infants

Reference	Country	Comparison methods	Inclusion/ Exclusion	Number (sex)	Number of tests	Age at measurement	Results
Animal and Animal Tissues							
Accuracy studies							
Sainz et al. (2003) [17]	The US	ADP, chemical analysis (CA), hydrostatic weighing	Bovine tissue with wide ranges of mass (1.39–9.95 kg) and FM% (2.08–34.40%)	24 bovine tissue phantoms (NA)	NR	NA	%Fat: ADP: 18.55% CA: 18.59% SD %fat ADP: 0.70% CA: 0.73% 95% LoA: −1.22-1. 13 %Fat
Frondas-Chauty et al. (2012) [44]	France	ADP, chemical analysis	Live piglets with wide ranges of mass (1.03–8.49 kg) and FM% (3.2–16.4%)	34 live piglets	NR	2, 7, and 21 days	%Fat: Pea Pod, 8.01 ± 4.03%; CA, 8.7 ± 4.1% Standard error of the estimate: 1.7% R^2^: 0.83
Reproducibility studies							
Frondas-Chauty et al. (2012) [44]	France	ADP within a day	Live piglets (aa above)	34 live piglets	Four times	2, 7, and 21 days	CV: ranging from 2.2 to 44.1% Root mean square CV: 17.9% CV values and median differed by age and %fat assessed using CA, with less variability in piglets with higher %fat
Full-Term Infants							
Accuracy studies							
Ma et al. (2004) [18]	The US & China	ADP, deuterium dilution	Healthy f ull-term infants (mixed ethnicity; white and Chinese)	53 (28F, 25 M)	2 days and 2 tests on first day	5.8 (6.0) (0.4–24.4) wks	%fat Pea Pod: 20.32–6.87%; deuterium: 20.39–6.68 95% LoA: − 6.84-6.71% (not related to body mass)
Ellis et al. (2007) [45]	The US	ADP, 4-CM	Full term healthy infants (mixed ethnic groups; white, African American, Hispanic American)	49 (24F, 25 M)	Twice	8.0 (5.4) wks	%Fat Pea Pod: 16.9 (6.5), 4-CM: 16.3 (7.2) (*P* = 0.62) 95% LoA: − 6.8-8.1%
Fields et al. (2012) [49]	The US	Pea Pod, DXA	Full term singleton infants, average for gestational age and with gestational age > =37, staying in hospital less than 3 days after delivery. nfants with congenital malformation were excluded. (ethnicity nor reported)	84 (47F, 37 M)	NR	6 mo; 168.4 (3.8) days	%Fat Pea Pod: 26.7 (4.7), DXA: 31.3 (3.6), (*P* < 0.001)
Reproducibility studies							
Yao et al. (2003) [16]	The US	ADP within and between-days – data from multiple compartment studies of Butte et al. and Fomon et al	Full term healthy infants with weight ranging between 3.40–7.45 kg and age ranging between 1.40–21.70 weeks (ethnicity not reported)	17 (8F, 9 M)	2 days and 2 tests per only one day	1.40–21.70 weeks (all 3 tests);	%fat: ranging 7.49–32.08% SD %Fat within day: 0.52; between day: 0.60 CV %fat within day: 2.85; between day: 2.95 95% LoA within day: − 2.0-1.2%; between day: − 2.2-1.7% (not a function of behavioural state)
Ma et al. (2004) [18]	The US & China	ADP within and between days	Healthy full-term infants (mixed ethnicity; white and Chinese)	36 (16F, 20 M)	2 days and 2 tests on first day	7.6 (7.2) (0.4–21.7) wks	SD %fat within day: 0.69%; between day: 0.72% CV %fat within day: 4.94; between day: 5.10% 95% LoA within day: − 2.7-3.1%; between day: − 2.9-1.9 (not a function of body f atness)
Ellis et al. (2007) [45]	The US	ADP within day	Full term healthy infants (mixed ethnic groups; white, African American, Hispanic American)	31 (18F, 13 M)	Twice	6.0 (3.3) wks	SD %fat within: 0.7% CV %fat within: 7.9% 95% LoA: − 2.3-3.1%
Pre-Term Infants							
Accuracy studies							
Roggero et al. (2012) [50]	Italy	Pea Pod, deuterium dilution	Preterm singleton infants with gestational age = < 36 weeks were included. Also term infants with gestational age > =37 were included. Infants aged > 1 mo of age, with congenital diseases, chromosomal abnormalities, respiratory distress syndrome, or severe brain, metabolic, cardiac, or gastrointestinal diseases were excluded.	10 (4F, 6 M)	NR	< 1 month	%fat Pea Pod: 5.67 (1.84), deuterium dilution: 5.99 (2.56), *P* = 0.53 95% LoA: −3.4-2.76% (not a function of mean values)
Forsum et al. (2016) [51]	Sweden	Pea Pod, deuterium dilution	Infants without sepsis or malformations born between 32 and 37 gestational weeks (ethnicity not reported)	14 (4F, 10 M)	NR	3–7 days old	%Fat Pea Pod: 4.2 (3.9), deuterium dilution 3.2 (3.8) (*P* > 0.05) 95% LoA: − 6.8-4.8% FFM density by Pea Pod did not correlate with those of deuterium dilution (r^2^ = 0.04, P > 0.05) Hydration factor: 83%
Reproducibility studies							
Roggero et al. (2012) [50]	Italy	NA	Preterm singleton infants with gestational age = < 36 weeks were included. Also term infants with gestational age > =37 were included. Infants aged > 1 mo of age, with congenital diseases, chromosomal abnormalities, respiratory distress syndrome, or severe brain, metabolic, cardiac, or gastrointestinal diseases were excluded.	Precision: 57 (29F, 28 M) Inter-devise reproducibility: 9 full term and 3 preterm (6F, 6 M)	Twice	< 1 month	%Fat SD: 1.1 95% LoA: − 2.05-2.36% **Inter-device reproducibility:** %fat test 1: 8.97%, test 2: 8.55% 95% LoA: − 1.87-2.71

*NA*, not applicable; *ADP*, air displacement plethysmography; *CA*, chemical analysis; *SD*, standard deviation; *LoA*, limits of agreement; wks, weeks; *US*, united states; *CV*, coefficient of variation; *4-CM*, 4 compartment model; *DXA*, dual energy X-ray absorptiometry

### Accuracy and reproducibility of pea pod

#### Studies using animal tissues and/or live animals

##### Accuracy

As part of the development of Pea Pod, Sainz and colleagues [17] were the first to evaluate the accuracy of Pea Pod against chemical analysis and hydrostatic weighing of 24 bovine tissue phantoms, and to assess its potential use in paediatric body composition assessment. Tissue phantom mass (1.39 to 9.95 kg) and fat content (2.1% to 34.4%) approximated that of infants between birth and six months of age. Compared with chemical analysis, the bias for FM% was − 0.04% and was constant across the FM% range (indicating no systematic bias), and the 95% limits of agreement were − 1.22% to 1.13%. Frondas-Chauty et al. [44] compared Pea Pod measures of FM% with biochemical analysis in 12 piglets at 2 to 21 days of age (weight range 1.03 to 8.49 kg). Bland-Altman analysis showed 95% limits of agreement from − 4% to 3%.

##### Reproducibility

Frondas-Chauty et al. also evaluated the reproducibility of Pea Pod in piglets (four measurements within one hour) [44]. Reproducibility was limited at low FM% (CV 14% to 32% at mean FM% of 3.7%) but better at higher FM% (CV 4% to 10% at mean FM% of 11.7%).

In summary, these studies suggest that Pea Pod has reasonable accuracy and reproducibility for FM% but reproducibility may be poor with lean body composition.

#### Studies in full-term infants

The accuracy and reproducibility of Pea Pod was examined in full-term infants by three studies each. All studies included both male and female infants. The accuracy studies included between 49 to 84 infants and the reproducibility studies from 17 to 36. The chronological age ranged from 0.4 to 24.6 weeks. The behavioural states [16, 18] and ethnicity [18, 45] were considered in two studies.

##### Accuracy

Ma et al. [18] examined the accuracy of Pea Pod measures of FM% against deuterium dilution in full-term infants aged 0.4 to 24.4 weeks and weighing 2.7 to 7.4 kg. Limits of agreement (95%) for FM% were relatively wide (− 6.84% to 6.71%). The authors reported that the results were not influenced by infant behavioural state. Ellis et al. [45] evaluated Pea Pod against a four-compartment model in full-term infants at 2 to 17 weeks of age. There was no bias for FM% but the 95% limits of agreement were also wide (− 6.8% to 8.1%). In multiple regression analysis that included protein and hydration fractions, the mineral fraction of FFM was the only significant factor associated with differences in FM% between Pea Pod and the four-compartment reference model (R^2^ 16%, *P* = 0.004). Fields et al. [49] compared Pea Pod and DXA in 84 full-term infants at 6 months of age. There was bias for FM% of − 4% and 95% limits of agreement of − 8% to 0%. However, the bias was positively related to FM%, suggesting underestimation of FM% in infants with lower fat content.

##### Reproducibility

The reproducibility (within and between days) of Pea Pod was examined in three studies [16, 18, 45]. Yao et al. studied 17 full-term infants aged between 1.4 to 21.7 weeks and reported that reproducibility was not affected by the infants’ behavioural state, body weight, urination, defecation, or FM% [16]. The within and between day 95% limits of agreement for FM% were − 2.0 to 1.2 and − 2.2 to 1.7, respectively. Similarly, Ma et al. found that in infants aged between 0.4 to 21.7 weeks, 95% limits of agreement for between day differences in FM% were − 2.9% and 1.9%, and the within subject CV for FM% was 4.9% [18]. Reproducibility was not influenced by infants’ behavioural state (awake and active, *N* = 147; crying intensely, *N* = 74; urination during the measurement, *N* = 33) or ethnicity (Asians, *N* = 20; white, *N* = 16). Finally, Ellis et al. [45] reported within day 95% limits of agreement for FM% of − 2.3% to 3.1% in 31 infants at a mean age of 6 weeks.

In summary, Pea Pod has reasonable reproducibility for FM% in full-term infants but only modest accuracy, with overestimation or underestimation of FM% by 6% to 8%. Although one DXA study suggested that Pea Pod systematically underestimates FM%, this not seen when referenced to other criterion measures. Studies included very few infants in the first week of life, so accuracy and reproducibility of Pea Pod in early neonatal period is unclear. Further, there are no data on the reproducibility and accuracy specifically in infants born small, large or appropriate for gestational age.

#### Studies in pre-term infants

##### Accuracy

Only two studies have specifically evaluated the accuracy of Pea Pod in pre-term infants, both using isotope dilution (Table 1) [50, 51]. Roggero et al. referenced Pea Pod with a two-compartment model and included 10 pre-term infants (≤36 weeks) aged less than one month with mean (SD) weight of 1.83 (0.21) kg [50]. The 95% limits of agreement for FM% were − 3.4% to 2.8% with no bias [50]. Forsum et al. [51] measured 14 pre-term infants at 32 to 35 weeks of gestation in the first week with a mean (SD) weight of 2.04 (0.33) kg. Compared to a three-compartment model, the 95% limits of agreement were relatively wide (− 6.8% to 4.8%) but there was no bias. This study also showed that at higher FFM density, Pea Pod underestimated FFM density.

##### Reproducibility

Roggero et al. [50] performed two consecutive Pea Pod measurements in 57 infants and the 95% limits of agreement were − 2.05% and 2.36%. They also compared measurements in 12 infants (3 preterm) using two Pea Pod devices in the same room and the inter-device 95% limits of agreement were − 1.87% and 2.71%.

In summary, there are few data on the validity of Pea Pod in preterm infants, but performance appears to be similar to full-term infants, that is, reasonable reproducibility but only modest accuracy.

### Practical challenges of pea pod

Despite the above concerns about accuracy, Pea Pod has been considered a valuable tool with a broad clinical research application. We identified 74 papers utilising Pea Pod to answer research questions, including studies comparing different clinical groups (pre-term vs. full-term infants, small for gestational age vs. appropriate for gestational age, different ethnicities, different feeding methods and breast milk compositions, and different health status or health indicators) [33, 52–69], assessing growth charts, to cross-validate FM values obtained by other techniques and anthropometric measures (e.g. skinfold) [70–76], monitoring growth patterns [54, 77–80], investigating the relationship between maternal and prenatal factors and health outcomes during infancy [81–86], and others [87]. The majority of these studies (*n* = 43) included only full-term infants [33, 52–54, 56–58, 60, 62, 66, 67, 69–72, 74, 75, 78, 80, 81, 83–86, 88–106], 13 only pre-term infants [9, 68, 73, 77, 79, 107–112], and 17 both full- and pre-term infants [55, 59, 61, 63–65, 76, 82, 87, 113–120]. Although, the use of Pea Pod in both full-term and pre-term infants and in specific clinical risk and ethnic groups is feasible, it might be associated with some practical challenges. Herein, we highlight and discuss potential challenges associated with the use of Pea Pod.

Measuring body composition in Pea Pod involves the simultaneous weighing and measuring of length and body volume. Pea Pod assesses body composition considering an infant’s anthropometric and demographic characteristics that are entered in to the Pea Pod system by personnel. As weight changes dramatically during early infancy, this information should be obtained consecutively with the Pea Pod test. Per the manufacturer manual, it is advised to conduct the Pea Pod test in duplicate to potentiate the accuracy. Repeat measures in infants might be associated with practical challenges and might require a third test which increases both researcher and caregiver burden. Out of 73 papers utilising Pea Pod, 13 reported repeated measurements, six of which reported duplicate tests [62, 67, 76, 85, 112, 114], six reported a third test if the results of the first two differed significantly [81, 102, 105, 116, 117, 120], and one reported three tests (performed on all subjects) [115], and the remaining studies that did not duplicate did not provide a rationale.

Furthermore, body moisture, temperature, and hair have been shown to significantly influence FM%, and underestimate it by 2% [121, 122]. Thus, Pea Pod testing should always be conducted at a moisture free and normal body temperature prior to measurement and in a resting state while wearing wig cap or smoothed hair using baby oil. Reproducibility studies have not suggested any effect of infant movement, crying, urination and defecation on the body composition measurements [18, 45], although the data are few.

The calibration process requires the placement of a hollow cylinder with known mass and volume into the Pea Pod. Mass and volume calibration should also be adjusted for any objects attached to the infants, which is mainly true for those remaining in hospital. Objects such as name bracelets and cord clamps affect body mass and volume readings and consequently FM and FFM. Some but not all studies reported adjusting for these objects [53, 58, 72, 76, 80, 89, 90, 98, 108]. Ramel et al. [108] also adjusted for other objects such as nasogastric tube and oximetry monitor.

Assessment of body composition by Pea Pod is not suitable for all infants. For example, Pea Pod is limited to infants weighing from 1 kg to 10 kg and with body volume > 1.85 L [14]. Infants who require oxygen, intravenous fluids and who are unstable also cannot easily undergo Pea Pod measurement. The inclusion/exclusion criteria of a large number of papers are based on these characteristics such as weight [9, 43, 55, 65, 71, 73, 78, 79, 88, 93, 108–111, 113, 114] and health condition [9, 33, 54, 61, 76, 77, 98, 100, 112, 123]).

Pea Pod accommodates infants weighing up to 10 kg (approximately six months of age), but the child and adult version of ADP (Bod Pod) only houses children aged ≥2 years. Unfortunately, use of ADP for the measurement of body composition in children aged 6 to 24 months has been shown to be inaccurate [124]. This leaves a large gap, from 6 months to 2 years, where body composition cannot be determined using ADP method. This is an important consideration for longitudinal research projects and may limit the applicability of ADP.

When infants with very low body fat content are measured in Pea Pod, the system may give errors and even a negative body fat content that is not physiologically plausible. Low body fat or negative body fat errors were reported in three studies [43, 80, 88], two of which were from Ethiopia [42, 76], in which 30 and 14 infants had to be excluded, respectively, and one from Australia [80], in which one infant was excluded. Similarly, spuriously high body fat content has also been reported in two studies [76, 80]. Carrberry et al. [80] reported excluding one infant – whose father was of Tongan ethnicity – due to a very high FM%, and Villar et al. [76] excluded three infants due to high FM% or FFM% (> 3 SD). These findings highlight the importance of considering measurement errors in preliminary sample size calculation by study investigators, particularly if a multi-ethnic population is under the investigation.

ADP devices operate under changing air temperature; for example, as an individual enters the test chamber the air temperature in the test chamber increases, resulting in different compressibility (adiabatic). However, the air in the thoracic cavity and near the surface of skin is isothermic and thus is more easily compressed (40%) than an equivalent volume of adiabatic air [125], which can affect FM readings. In adult ADP devices, thoracic gas volume is measured directly, but this is impractical in infants and young children. Thus, the thoracic gas volume is predicted based on infant weight, length and age. The prediction equation has been validated in full-term infants [126, 127], but its accuracy in pre-term infants and those with lung disease is unknown. Accordingly, Pea Pod measurements in infants with lung disease should be interpreted with caution because of altered thoracic gas volume.

To account for the skin surface artefact, the system software applies a correction factor based on body surface area. Body surface estimates are less accurate at the extremes of body size [128]. This might have implications for studies involving pre-term and growth restricted infants, and those born large for gestational age, such as with maternal diabetes.

The Pea Pod system calculates FM from body weight, volume and the density of FM and FFM. It is well documented that the density of fat mass is constant at 0.9007 g/ml, though the density of FFM varies with age and physiological state. The FFM density estimates use in Pea Pod are derived from full-term infants [129]. Fomon et al. based their model on the measurements of protein, minerals and water in FFM [129], and Butte et al. on the measurements of body weight, body protein, bone minerals, and body water [130]. An important difference between these two models is the estimated amount of water (hydration factor) in FFM, which is lower in the Fomon model, specifically in the neonatal period (80.6% for both boys and girls at birth; 82.7% for boys and 83.1% for girls at two weeks, respectively) [129, 130]. Thus the Fomon model may produce FM values that are greater than those obtained using the Butte model. For example, Eriksson [91] reported a greater increase in FM% between one and 12 weeks of age when using the Butte model than the Fomon model. Consideration of the FFM model is important in longitudinal research i and when comparing results from different studies.

The hydration factor of FFM changes considerably during early infancy (particularly in pre-term infants) and can affect estimates of FFM and FM [131]. The Pea Pod system has algorithms to account the fluctuation in hydration in this period of life [51]. However, Forsum et al. [51] found that when compared to a three compartment model using isotope dilution, Pea Pod gave biased values for predicted FFM density in moderately pre-term infants (lower by 0.0019 ± 0.0058 g/ml). Biological variability in hydration factors may lead to variations in FFM density and consequently inaccurate body composition readings.

### Conclusion and future lines of research

Pea Pod is a convenient a way to measure body composition in infants and may be useful for monitoring groups of infants but appears to have only modest accuracy in individual subjects. Multiple factors may affect accuracy, including body moisture and temperature, objects attached to infants, extremes of body size, thoracic gas prediction equations, FFM density, and fluctuations in FFM hydration factor. There are few data on the accuracy of Pea Pod in preterm infants and a reference body composition for pre-term infants is lacking. Further, the validity of Pea Pod in different clinical risk groups and ethnicities is unknown. Given these gaps in the current literature, the following are suggested as future lines of research:

1. Further validate pea pod in infants against the gold standard four compartment model, especially in pre-term infants and in more diverse populations.
2. Investigate the accuracy/reproducibility of Pea Pod in different birth weight for gestational age categories.
3. Investigate the impact of FFM hydration level and density in Pea Pod measurements.
4. Develop and validate specific prediction equations to estimate thoracic cavity gas in pre-term infants and more diverse infant populations.
5. Investigate the impact of body movement, urination, defecation, and lung disease on Pea Pod measurements.
6. Develop and design equipment working on the ADP principles that could accommodate children aged 6–24 months to support longitudinal research.
7. Develop a set of recommendations for complete and transparent reporting of study procedures when using Pea Pod.
8. Investigate the best body composition measure(s) that accurately predicts growth, nutritional status, obesity and health outcomes.
9. Establish body composition reference data or curves for both full-term and pre-term infants, such as FM/FFM for length z-scores.

## Abbreviations

4-CM: 4 Compartment Model
ADP: Air displacement plethysmography
BIA: Bioelectrical Impedance Analysis
BMI: Body Mass Index
CR: Chemical Analysis
CV: Coefficient of variation
DXA: Dual Energy X-ray Absorptiometry
FFM: Fat Free Mass
FFMI: Fat Free Mass Index
FM: Fat Mass
FMI: Fat Mass Index
LM: Lean Mass
LoA: Limits of Agreement
MRI: Magnetic Resonance Imaging
NA: Not Applicable
NR: Not Reported
SD: Standard Deviation
US: United States
